# T2D@ZJU: a knowledgebase integrating heterogeneous connections associated with type 2 diabetes mellitus

**DOI:** 10.1093/database/bat052

**Published:** 2013-07-11

**Authors:** Zhenzhong Yang, Jihong Yang, Wei Liu, Leihong Wu, Li Xing, Yi Wang, Xiaohui Fan, Yiyu Cheng

**Affiliations:** Pharmaceutical Informatics Institute, College of Pharmaceutical Sciences, Zhejiang University, Hangzhou 310058, China

## Abstract

Type 2 diabetes mellitus (T2D), affecting >90% of the diabetic patients, is one of the major threats to human health. A comprehensive understanding of the mechanisms of T2D at molecular level is essential to facilitate the related translational research. Here, we introduce a comprehensive and up-to-date knowledgebase for T2D, i.e. T2D@ZJU. T2D@ZJU contains three levels of heterogeneous connections associated with T2D, which is retrieved from pathway databases, protein–protein interaction databases and literature, respectively. In current release, T2D@ZJU contains 1078 T2D related entities such as proteins, protein complexes, drugs and others together with their corresponding relationships, which include 3069 manually curated connections, 14 893 protein–protein interactions and 26 716 relationships identified by text-mining technology. Moreover, T2D@ZJU provides a user-friendly web interface for users to browse and search data. A Cytoscape Web-based interactive network browser is available to visualize the corresponding network relationships between T2D-related entities. The functionality of T2D@ZJU is shown by means of several case studies.

**Database URL:** http://tcm.zju.edu.cn/t2d

## Introduction

Diabetes mellitus is one of the major threats to human health, which is expected to affect 552 million people by 2030 (1). More than 90% of the diabetic patients are affected with type 2 diabetes mellitus (T2D) (2). Although several mechanisms of T2D have been proposed, including metabolic overload, mitochondrial dysfunction, inflammatory mediators, deposition of toxic amyloid fibrils and etc, the pathogenesis of T2D is still under investigation (3, 4). Hence, a comprehensive understanding of the mechanisms of T2D at molecular level is urgently needed.

Nowadays, the existing knowledge of T2D is located in different formats at scattered places (5, 6). The published literature, as well as the protein–protein interaction (PPI) databases, provides a huge source of knowledge about T2D, whereas several pathway databases offer highly refined information of the molecule regulation processes. To integrate the existing knowledge, there are only a few attempts, such as T2D-Db and T2DGADB, to develop T2D-related databases. T2D-Db manually curated 330 candidate genes from the Pubmed literature and provided their corresponding information (6). T2DGADB collected 701 publications in T2D genetic association studies (7). Therefore, the development of a comprehensive database able to cover current understandings of T2D is in high demand.

Here, we introduce a comprehensive and up-to-date knowledgebase for T2D, i.e. T2D@ZJU. T2D@ZJU contains three levels of data associated with T2D, which is retrieved from pathway databases, PPI databases and literature, respectively. In comparison with the previous T2D-related databases, which focus on the information of the T2D-related genes, T2D@ZJU organizes the integrated information in a manner of network, which could be further expanded as desired with data of three distinguishable levels, in terms of data reliability and information coverage, to meet the needs of network pharmacology studies. To enhance the readability of these data, T2D@ZJU provides an interactive web tool for users to visualize the network relationships between T2D-related entities, which will offer a better understanding of T2D for basic and clinical researchers and hereby facilitate the T2D-related studies of network pharmacology and multi-target therapeutics.

## Contents and Constructions

**Figure 1.**
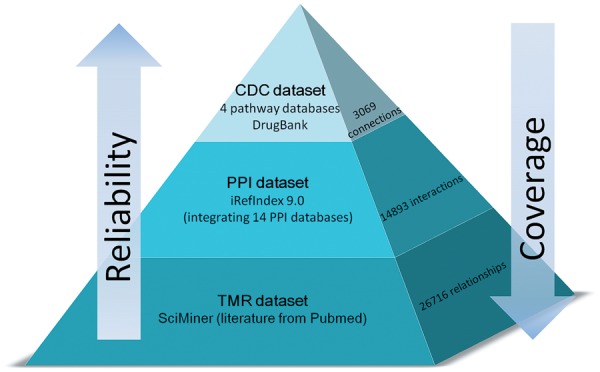
Overview of the T2D@ZJU, which is composed of three data sets, i.e. CDC data set, PPI data set and TMR data set.

As illustrated in Figure 1, in current release, T2D@ZJU contains three levels of data, namely, curated directed connections (CDC) data set, PPI data set and text-mining-based relationships (TMR) data set, which are complementary owing to the different levels of data reliability and information coverage. The web interface of T2D@ZJU has been developed using HTML/CSS, Javascript, Ruby on Rails and Cytoscape. All data in the database have been stored in SQLite tables.

### CDC data set

CDC data set was manually integrated from four well established pathway databases including KEGG (8), Reactome (9), BioCarta (http://www.biocarta.com) and PANTHER (10) as well as DrugBank (11). All the human T2D-related pathways in these databases were collected. Individual interactions are the compositions of the biological pathways. The concept and the boundary of pathway are always not coincident between different pathway databases. The aim of establishing CDC data set was to construct a network derived from the pathway databases. Therefore, individual interactions from different databases were compared with each other and adopted to construct CDC data set. Moreover, novel loci associated with T2D newly identified by genome-wide association studies and other association studies were also curated and integrated to CDC data set. In total, 1078 T2D-related entities (including 885 proteins, 14 protein complexes, 36 drugs and 143 small molecules) and 3069 directed connections are collected and imported to CDC data set.

### PPI data set

iRefIndex 9.0 (12), an integrated PPI source containing 14 PPI databases, served as the source of the PPI data set. After downloading iRefIndex 9.0, the human interactions associated with the T2D-related proteins in CDC data set were extracted from it. The classes of the PPI (e.g. experimentally validated, predicted, etc.) along with the specific detection methods, interaction types and PubMed Identifiers (PMIDs) of the origin literature were also labeled. The PPI data set contains 14893 PPIs in current release.

### TMR data set

The relationships among the T2D-related proteins were further investigated by a text-mining tool, i.e. SciMiner (13). SciMiner was run on a query of ‘type 2 diabetes’ and found 53 960 documents as of 31 December 2012. SciMiner uses a dictionary and rule-based approach to recognize biomedical objects (13). The documents with less than two genes or proteins found were removed. The remained documents were used to acquire the relationships. It is considered as a TMR if two proteins were co-occurred in at least one article. The TMR data set so far contains 26 716 relationships associated with the T2D-related proteins in CDC data set. In this work, frequency of the proteins co-occurrence was counted to index the weight of the relationship. The PMIDs of the origin literature were also labeled.

### T2D network

**Figure 2.**
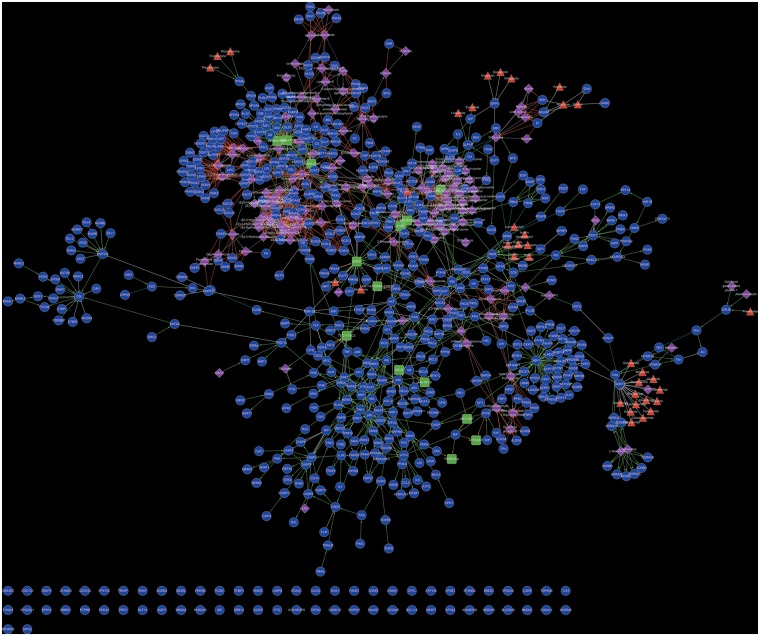
The T2D network constructed on the basis of CDC data set. In the network, blue circle, round rectangle, purple diamond and red triangle represented protein, protein complex, small molecule and drug, respectively.

T2D@ZJU provides a Cytoscape Web (14) based interactive network browser for users to visualize the network relationships between T2D-related entities. The T2D network constructed on the basis of CDC data set is shown in Figure 2. To improve the readability of the network, entities that play same mechanistic role within the network were aggregated into an entity class, e.g. the entity class ‘AKT’ includes AKT1, AKT2 and AKT3.

## Utilities

**Figure 3.**
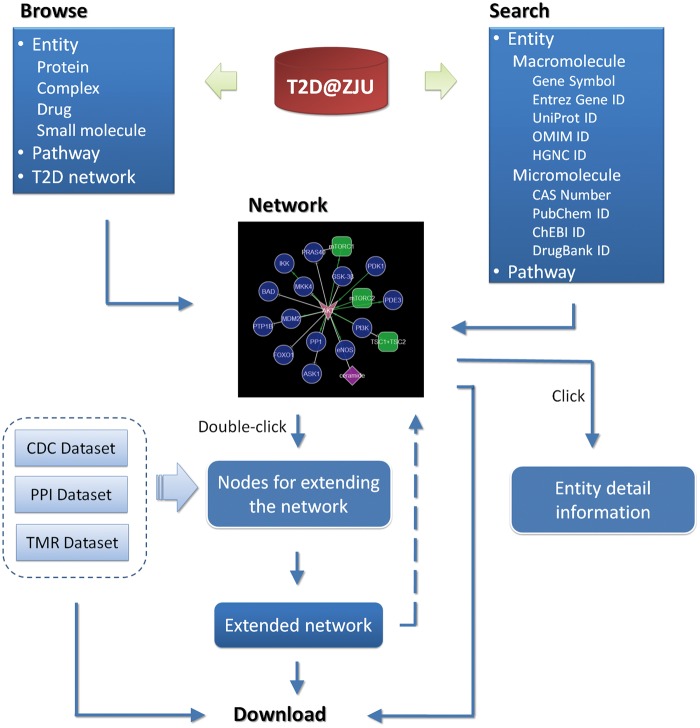
Graphical summary of the main functions of theT2D@ZJU web interface.

T2D@ZJU is freely available to academic users, which provides a user-friendly web interface for users to browse and search data. The main functions of the web interface are illustrated in Figure 3.

### Search

The query interface of T2D@ZJU allows users to perform two types of searches. Users can query using entity/entity class names or external identifiers (e.g. gene symbol, Entrez Gene ID, UniProt ID, etc.) in search box. Once given a query, the background program can find all the directly related entities/entity classes and draw the corresponding networks. Information of all the connections in the network is listed below the network, and the PMIDs of the references are linked to the respective literature sources. Clicking on any node of a given network, the detailed information would be listed with external links. Users also can search the pathway associated with T2D by selecting a pathway name in the dropdown box, and all the connections related to the corresponding pathway will be returned.

**Figure 4.**
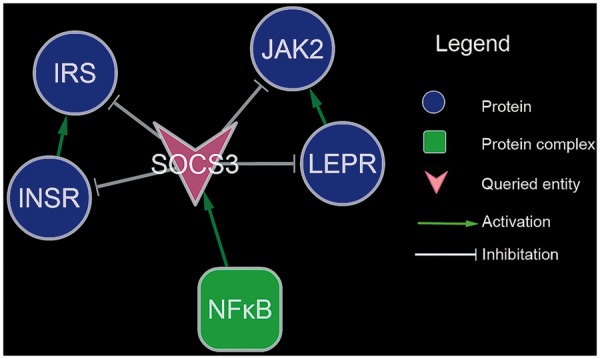
The outcome of the search with ‘SOCS3’ as the query keyword.

To demonstrate the utility of the search function and the associated knowledge discovery, a use case was applied. A search with ‘SOCS3’ as the query keyword resulted in five entities (INSR, IRS, LEPR, JAK2 and NFκB, respectively) related to SOCS3 directly. As displayed in Figure 4, these six entities formed a network through seven interactions. In obesity, SOCS3 is upregulated in the skeletal muscle, liver, adipose tissue and hypothalamus (15–18), which is associated with increases in inflammation. SOCS3 is a major negative regulator of insulin and leptin signaling (15), which is thought to contribute to the pathogenesis of insulin resistance. In adipocytes, SOCS3 deficiency increases insulin-stimulated IRS1 and IRS2 phosphorylation, whereas overexpression of SOCS3 reduces both IRS1 protein levels and the phosphorylation of IRS1 and IRS2 (19). Genetic deletion of SOCS3 in mouse liver results in increased insulin sensitivity owing to increased IRS1 phosphorylation (20). SOCS3 has also been reported to co-immunoprecipitate with both INSR and IRS1 in skeletal muscle (21). SOCS3 inhibits leptin signaling by binding to phosphorylated tyrosine residue of the leptin receptor (22). SOCS3 is a major regulator of JAK signaling, which binds and inhibits the catalytic domains of JAK2 via an evolutionarily conserved motif unique to JAKs (23).

Therapies aimed at inhibiting SOCS3 in skeletal muscle may be a favorable strategy to reverse glucose intolerance and insulin resistance (24). NFκB plays an important role in regulating PIM2 induced SOCS3 expression (25). Therefore, inhibiting NFκB might be an alternative way to reduce SOCS3 expression. Moreover, Salicylates have been reported to inhibiting NFκB by inhibiting IκB kinase-β (26–28). Reduce of SOCS3 expression might be involved in the mechanism of the anti-inflammatory drug through which it works.

### Browse and network extension

Users are allowed to browse the T2D networks. Moving the mouse over any node of the network, all the directly related interactions will be highlighted, whereas clicking on a node, details about the node will be listed below. Double-clicking on any node of a given network, all the dirctly related entities from different data sets (CDC, PPI or TMR data set) will be listed, and users are able to select any of listed entities to extend the network.

**Figure 5.**
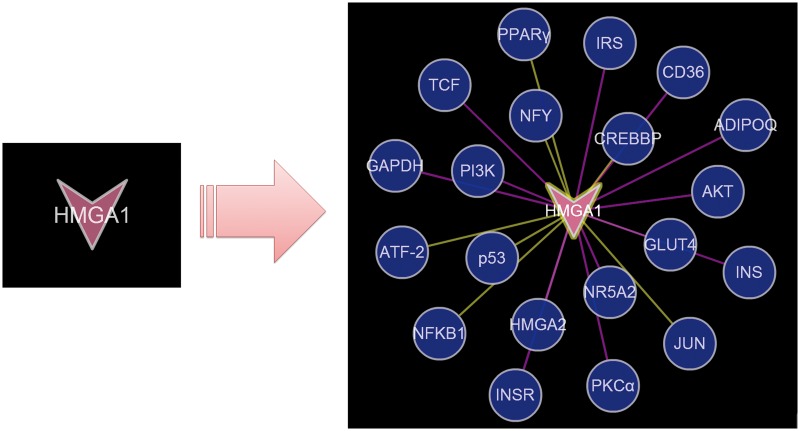
The extending of HMGA1 with PPI and TMR data sets. The yellow edges represented relationships derived from PPI data set, whereas the pink represented relationships from TMR data set.

Nodes for which knowledge is still limited have fewer connections with other entities in the T2D network. However, some of them are proposed to be the associated with enhanced risk of T2D, such as HMGA1 (29,30). Connections from PPI and TMR data sets provide valuable clues, as shown in Figure 5, allowing researchers to propose hypotheses that can be tested by further experiments.

### Download

All three data sets are freely available at the download page. We also provide an illustration and examples to guide the users through the different functions of the web interface at the help page.

### Other application

**Figure 6.**
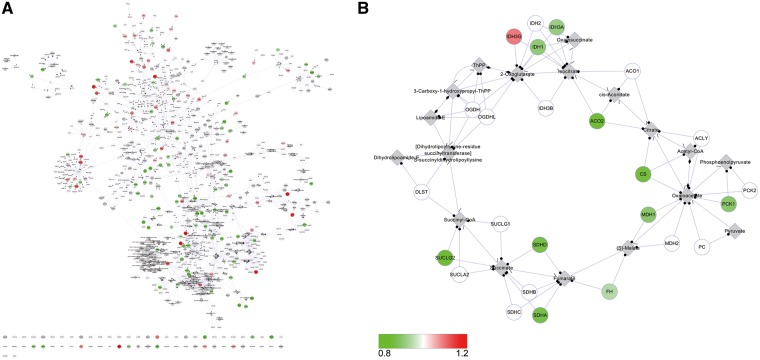
Visualization of transcriptomics data on the T2D network. (**A**) The gene expression status of the T2D network. (**B**) The expression of genes involved in citrate cycle. The fold changes of the gene expression were depicted by colors, as indicated by the bar. The networks were constructed in Cytoscape.

T2D@ZJU could also offer a view of T2D for visualization of ‘Omics’ data, such as transcriptomics data and proteomics data. For instance, users could download the T2D network and map the ‘Omics’ data into it. To represent this functionality, we used transcriptomics data from van Tienen *et al.* (31) comparing skeletal muscle from longstanding T2D patients against that from controls matched by age and body mass index (Gene Expression Omnibus accession number GSE19420). The fold changes in the gene expression of the T2D group relative to the controls were projected onto the T2D network. The color of the node coded the difference of the gene expression. In detail, red and green represented up- and downregulation, respectively, whereas gray meant the data were lacking. The status of the gene expression was intuitively shown in Figure 6A. In general, fold changes were small. The expression of genes involved in citrate cycle was displayed in detail in Figure 6B. In the T2D group, distinctly, almost all the expression of genes involved in citrate cycle was decreased compared with controls. It demonstrated that the ability of using glucose as energy source was lower in T2D patients, which was consistent with the previous understanding (32).

## Conclusions

In summary, T2D@ZJU provides a comprehensive knowledgebase about T2D, including a three levels layout on the associated connections. It builds a basis for the further study of T2D in system biology, which helps to better understand of the complex disease, as well as give clues facilitating researchers to propose testable hypotheses. Furthermore, the T2D-related studies of network pharmacology and multi-target therapeutics would also be accelerated.

The T2D associated information is expected to grow with the continuing effort of the researchers. Therefore, T2D@ZJU will be updated periodically. Specific network analysis tools would be developed for the further research. Semantic data mining is intended to be used to extract the Pubmed literature related to T2D to improve the confidence levels of the TMR data set.

## Funding

National S&T Major Project (No. 2012ZX09503001), Program for New Century Excellent Talents in University (NCET-12-0488) and Fundamental Research Funds for the Central Universities. Funding for open access charge: National S&T Major Project (No. 2012ZX09503001).

*Conflict of interest*. None declared.
